# Dentition

**Published:** 1859-08

**Authors:** J. P. H. Brown

**Affiliations:** Dentist, Atlanta, Georgia


### ARTICLE IX.

*Dentition.* By J. P. H. BROWN, Dentist, Atlanta, Georgia.

The process of Dentition may be divided into three stages, first—The *papillary*, which commences with the formation of the primitive dental groove at about the sixth week of fœtal life, and terminates when the papilla are completely hidden by the closure of the mouths of the follicles, and of the groove itself. Second—The *sacular*, includes the period when the follicles are shut sacks, and the papilla pulps; and when the whole formation of the dentine and enamel takes place. The third stage is the *eruptive*, and embraces the completion of the teeth, their passage through the gum, the shedding of the first set, and also the eruption of the second set. To this stage of dentition I purpose confining my remarks.

It is a puzzle with physiologists to know exactly *how* the evolution of a tooth from its alveolar cell, through the gum, is effected. This process, no doubt, results from the lengthening of the fang by a deposition of new dentinal substances, so that the crown is made to press upon the sack; and also from the contraction of the sack itself. The eruption of the temporary teeth varies very much in regard to time, with the health and constitution of the child. The following order may be considered a fair average ; the lower teeth usually preceding the upper by a short interval : the two central incisors at the 7th month ; the lateral incisors the 9th month ; the first molars the 14th month ; the cuspidati the 18th month, and the second molars at the 24th month. Between the cutting of each of these classes of teeth there seems to be a period of repose, when nature rests as it were, from her laborious efforts. Thus between the eruption of the central and lateral incisors there is a space of two months ; between the first molars and the cuspidati, four months, and between these last and the second molars, six months. In weaning, advantage should always be taken of these intervals, for then the irritability of the system has in a great measure subsided, and the infant is better prepared for the transition from the pabulum of the mother's breast to more solid and substantial food.

Dentition is not of itself a morbid process nor a disease, but a purely physiological act ; for when it takes place in a natural and healthy state of the system, no inconvenience or symptoms of disease whatever, are experienced. In the animal creation, we see no pain or difficulty arising from the eruption of the teeth in the young ; and thus it would be with the *human animal* if the laws of health were strictly observed. The *direct* or exciting cause of painful dentition, is the pressure of the teeth upon the gum ; but the *indirect* or pre-disposing cause must be looked for in the constitution of the child. The rapid development of any organ requires a certain amount of constitutional vigor to accomplish it.

Now, many children are born into the world with such feeble physical powers, that when any demand is made upon them, either by accidental or physiological causes, they readily yield to the action of the irritant, or morbific agents. While others, though born with sufficient stamina, have their systems in a weak and irritable condition, brought about by bad diet, impure air, and improper management. To such infants the period of teething is one of great risk and imminent danger.

One of the earliest symptoms of morbid dentition is manifested by the child's grasping the nipple more loosely than ordinarily ; frequently letting it go, and crying, as if pained by the effort at nursing. The saliva flows in larger quantities than usual, from tne mouth ; the gums, which were at first unaltered, begin to swell, and become inflamed and painful, and seem to be relieved by rubbing them. The mouth becomes hot ; and where the tooth is going to present itself, a pale or bright red elevated spot appears upon the gum, which is very painful when pressed upon. These local disorders are always accompanied, in a greater or less degree, by some of the general symptoms of diarrhœa, vomiting, costiveness, pyrexiæ, eruptive affections of the skin, spasms of the glottis, convulsions, etc. There are many intractable infantile ailments which are augmented by morbid dentition, but do not owe their origin to it.

In all these cases of abnormal teething two main indications of treatment are to be observed : First, to allay local irritation, and, second, to alleviate constitutional symptoms and support the strength. Rubbing the gums well and frequently, with the finger of the mother or nurse, greatly assists in allaying the local excitement. In fact, this simple means, in connexion with judicious general treatment, is often sufficient. But when not, the gums should be lanced freely. For this purpose it is very necessary to have a proper instrument. The blade should be somewhat rounded, rather broad, and possess a keen edge. The incision should be inclined outward in order to avoid injuring the tissues which connect the permanent with the temporary teeth. The gums must be cut down until the lancet impinges upon the approaching tooth—the superficial incision will not answer. When a molar tooth is in question a crucial incision may at times be necessary.

In the eruption of the permanent or second set of teeth, very little local irritation or general disturbance is experienced. Though sometimes the cutting of the *dentes sapientiæ* gives rise to considerable trouble, particularly when the arch of the jaw is contracted, and they have deviated from their natural position. They often cause violent neuralgic pains in different parts of the face; inflammation and swelling of the surrounding parts; and frequently, abscess. The writer has attended several cases of abscess of these parts when neither patient nor medical attendant suspected the cause. The gums are often inflamed, thickened and callous over the teeth, and should be freely lanced. Sometimes nothing short of extraction will suffice; and this operation should always be resorted to when they are crowded and occupy a misplaced position.

